# Ligands of CD6: roles in the pathogenesis and treatment of cancer

**DOI:** 10.3389/fimmu.2024.1528478

**Published:** 2025-01-07

**Authors:** Mikel Gurrea-Rubio, Feng Lin, Max S. Wicha, Yang Mao-Draayer, David A. Fox

**Affiliations:** ^1^ Division of Rheumatology, Department of Internal Medicine, University of Michigan, Ann Arbor, MI, United States; ^2^ Department of Immunity and Inflammation, Lerner Research Institute, Cleveland Clinic, Cleveland, OH, United States; ^3^ Department of Internal Medicine, University of Michigan, Ann Arbor, MI, United States; ^4^ Multiple Sclerosis Center of Excellence, Oklahoma Medical Research Foundation, Oklahoma City, OK, United States

**Keywords:** CD6, ALCAM (CD166), CD318, CD44, autoimmune disease, cancer

## Abstract

Cluster of Differentiation 6 (CD6), an established marker of T cells, has multiple and complex functions in regulation of T cell activation and proliferation, and in adhesion of T cells to antigen-presenting cells and epithelial cells in various organs and tissues. Early studies on CD6 demonstrated its role in mediating cell-cell interactions through its first ligand to be identified, CD166/ALCAM. The observation of CD6-dependent functions of T cells that could not be explained by interactions with CD166/ALCAM led to discovery of a second ligand, CD318/CDCP1. An additional cell surface molecule (CD44) is being studied as a potential third ligand of CD6. CD166, CD318, and CD44 are widely expressed by both differentiated cancer cells and cancer stem-like cells, and the level of their expression generally correlates with poor prognosis and increased metastatic potential. Therefore, there has been an increased focus on understanding how CD6 interacts with its ligands in the context of cancer biology and cancer immunotherapy. In this review, we assess the roles of these CD6 ligands in both the pathogenesis and treatment of cancer.

## What is CD6?

CD6, a type I transmembrane glycoprotein belonging to the highly conserved scavenger receptor cysteine-rich superfamily (SRCR-SF), is expressed by all developing and mature T-lymphocytes, a small fraction of mature B (CD5^+^ or B1a) cells, about one-half of human, but not mouse, NK (CD56^dim^CD16^+^) cells, and to a lesser extent, the basal ganglia and cerebellar cortex regions of the brain (1–3). CD6 is composed of an extracellular region consisting of three SRCR domains, a transmembrane region, and a long cytoplasmic tail containing phosphorylatable residues for intracellular signal transduction (4). Galectins, specifically Galectin-1 and -3, function as binding partners for CD6, primarily by interacting with specific carbohydrate moieties on CD6 (5), thereby modulating its function in immune cell adhesion, migration, and activation, particularly in the context of T cell signaling and immune response regulation (5).

**Figure 1:**
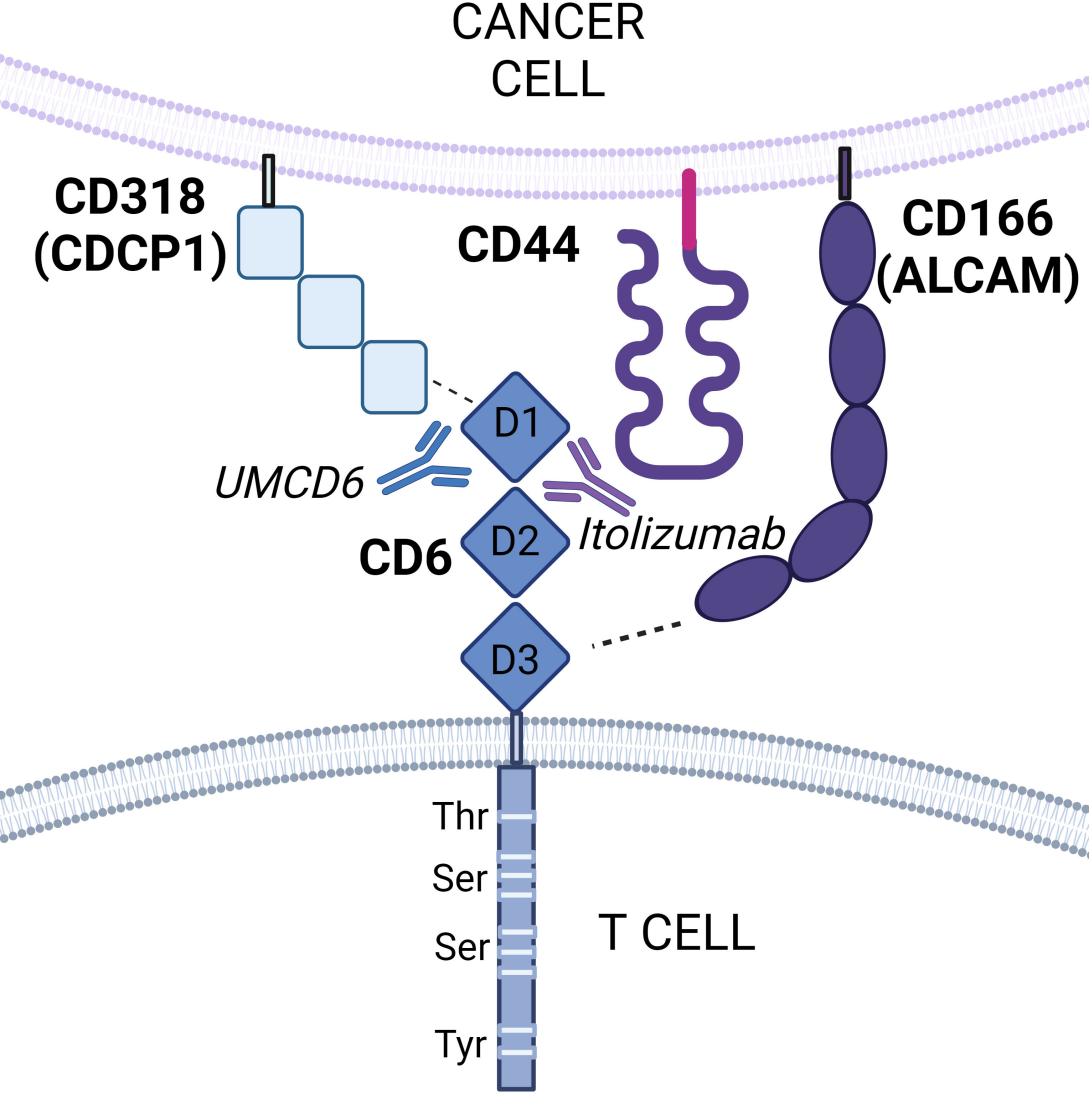
Schematic representation of CD6 interactions with its ligands. CD6 is composed of three extracellular scavenger receptor cysteine-rich domains (domains 1, 2, and 3) and an intracellular cytoplasmic that contains phosphorylatable residues for intracellular signal transduction. CD6 functions by interacting with its ligands CD166 (ALCAM), CD318 (CDCP1), and potentially CD44. CD166 is known to bind domain 3 of CD6, whereas CD318 binds domain 1. A third ligand, CD44, has been recently identified as a potential novel receptor for CD6 by proximity labeling. Both CD6/CD166 and CD6/CD318 interactions can selectively be targeted by the monoclonal anti-CD6 antibodies *itolizumab* and UMCD6 respectively.

Thus far, there are two mammalian CD6 ligands identified: CD166/activated leukocyte cell adhesion molecule (ALCAM) and CD318, also known as CUB domain containing protein 1 (CDCP1). Additionally, a new study by Borjini et al. shows strong biochemical and biophysical evidence that supports CD44 as a novel CD6 ligand (4, 6, 7) (**Figure 1**). All three CD6 ligands are widely expressed on the surface of various tissues, including endothelial and epithelial cells, antigen-presenting cells (APCs), and cancer cells.

## What is the function of CD6?

CD6 is known to play critical roles in T cell activation and signaling, cell adhesion, regulation of immune responses, and immune synapse formation (8, 9). Evidence that CD6 is involved in T cell activation was first suggested by Gangemi et al., who demonstrated that monoclonal antibodies (mAbs) targeting CD6 were able to induce T cell activation, T cell proliferation, and increase in IL-2 receptor expression (10). Since then, multiple studies have further substantiated a co-stimulatory role of CD6, enhancing T cell receptor (TCR) signaling when engaged (11–13). However, more recent studies suggest that CD6 can also act as an attenuator of early and late T cell responses in a ligand-independent manner (14, 15).

The involvement of CD6 in the immune synapse was first described by Gimferrer et al., whose studies showed that a fraction of CD6 molecules physically associate with the TCR/CD3 complex. Image analysis of Ag-specific T-APC conjugates further demonstrated that CD6 and its ligand CD166 colocalized with TCR/CD3 at the center of the immunological synapse. Moreover, their studies also found that addition of a soluble form of rCD6 reduces mature Ag-specific T-APC conjugates, indicating CD6 mediates early cell-cell interactions needed for immunological synapse maturation (16). Further studies conducted by Tudor et al. on CD6’s role in the immune synapse demonstrated that when CD166 binds CD6, a supramolecular complex is formed with the adaptor proteins ezrin and syntenin-1 coupling the cytoplasmic tail of CD166 to actin, stabilizing the immunological synapse during dendritic cell-T cell interactions (17).

CD6 also plays a significant role in cell adhesion. The sustained attachment of T lymphocytes to endothelial cells and antigen-presenting cells (APCs) is dependent on CD166 binding CD6, which also facilitates effective immune responses and cell migration to inflammatory sites in autoimmune disease. Importantly, CD166 participates in leukocyte migration across the blood-brain barrier (BBB), which is upregulated in active multiple sclerosis and experimental autoimmune encephalomyelitis (18). Furthermore, blockade of CD166 restricts the transmigration of CD4+ lymphocytes and monocytes across BBB endothelium, reducing the severity and delaying the onset of encephalomyelitis. CD6 also mediates adhesion of T cells to other tissue-specific cell types such as keratinocytes and synovial fibroblasts (19, 20). Such interactions involve engagement of both CD166 and CD318, which bind to distinct epitopes of CD6.

CD6 has also been shown to play a dual role in regulating immune responses: while it enhances T cell activation, it also has regulatory functions that help maintain immune homeostasis. Orta Mascaró et al. showed that CD6 can function as a negative modulator of TCR-mediated signaling. The molecular mechanisms by which CD6 restrains TCR signaling are not well described, as CD6 signaling pathway is still relatively unknown. However, given the association of CD5 and CD6 at the T cell surface, and the regulatory role of CD6 on CD5 Tyr phosphorylation, CD5 could mediate such a negative effect. CD6 signaling can therefore modulate the production of cytokines and other immune mediators, ensuring a balanced immune response that prevents excessive inflammation and autoimmunity (21). Recent studies on the CD6 signalosome, conducted by Mori et al., confirmed that the TCR-inducible CD6 signalosome had both positive (SLP-76, ZAP70, VAV1) and negative (UBASH3A/STS-2) regulators of T cell activation (22).

## Is CD6 linked to autoimmunity?

CD6 has been linked to autoimmunity, and CD6-targeting antibodies have been proposed as a promising therapy for several autoimmune diseases. Given that CD6 is required for optimal immune response, it is not surprising that many studies have linked CD6 with the pathogenesis of human autoimmune disease, such as Behcet’s disease (23), multiple sclerosis (MS) (24, 25), psoriasis (26), Sjögren’s syndrome (27, 28), rheumatoid arthritis (RA) (29), uveitis (30), inflammatory bowel disease (31), and most recently, lupus nephritis (32). For instance, CD6 has been identified and validated as a risk gene for MS (25), single-nucleotide polymorphisms (rs17824933, rs11230563 and rs12360861) are associated with severe forms of psoriasis (33), and high expression of CD6 has been observed in RA joints (20, 34). Importantly, in mouse models of MS and RA, in both *CD6^-/-^* mice and CD6-humanized mice treated with UMCD6, an anti-human CD6 monoclonal antibody targeting the CD6/CD318 interaction, striking reductions in clinical signs of disease, pathogenic Th1/Th17 responses and inflammatory cell infiltration into the target organs are observed (24, 29). Similarly, *CD6^-/-^* mice with experimental autoimmune uveitis (EAU), have decreased retinal inflammation and reduced autoreactive T-cell responses (30).

## Is there a role for CD6 in alloreactivity?

The role of CD6 in alloreactivity was described by Rasmussen et al., who discovered a subpopulation of peripheral blood T lymphocytes with low or no CD6 (CD3^+^CD5^int^CD6^lo/−^) in the blood of healthy volunteers. CD6^lo/−^ T cells showed decreased reactivity to allogeneic stimulation, but not to mitogenic lectins (PHA) or soluble recall antigens (tetanus toxoid) (35). Human natural regulatory T cells (nTreg) were later found to exhibit low/negative CD6 expression, suggesting that these cells could be the same CD6^lo/−^ population that Rasmussen et al. had discovered (36).

Additionally, CD6 is being studied for its role in graft rejection and tolerance. Rambaldi et al. recently examined expression of CD6 in patients after allogeneic cell transplantation and found that CD6 expression was reduced in Treg and CD8+T cells in acute graft-versus-host disease (aGvHD) compared to healthy donors. Itolizumab, an anti-CD6 mAb that targets the CD6/CD166 axis, inhibited CD4 and CD8 T-cell activation and proliferation in pre-GvHD samples, but inhibition was less prominent in samples collected after aGvHD onset. Modulating CD6 activity could therefore enhance immune tolerance to transplanted organs, reducing the risk of rejection and improving graft survival (37).

## Is CD6 expressed by lymphoid malignancies?

CD6 is expressed by many lymphoid malignancies. Zhao et al. found elevated expression of CD6 in aggressive NK-cell leukemia/lymphoma (ANKLL) and extranodal NK/T-cell lymphoma (ENKTL), suggesting that CD6 could be a therapeutic target for these hematological malignances (38). In patients with B-cell chronic lymphocytic leukemia (CLL), CD6 is also expressed on a significant proportion of their malignant B cells (39, 40). Parameswaran et al. recently confirmed that CD6 is indeed highly expressed on most T cell lymphoma cell lines. Importantly, a CD6-targeted antibody-drug conjugate (anti-CD6-ADC) selectively kills T cell lymphoma cells *in vitro* and reverses the development of tumors *in vivo* in mouse models of T cell lymphoma (TCL) (41).

## Is CD6 involved in solid malignancies?

The proteolytic cleavage of the CD6 extracellular region following lymphocyte activation gives rise to soluble CD6 (sCD6) (42), which is found in serum of cancer patients (43). Transgenic mice expressing high circulating levels of human sCD6 (shCD6LckEμTg) provided the first indication of the immunotherapeutic potential of shCD6 against cancer. These mice demonstrated decreased tumor growth when injected with subcutaneous syngeneic cancer cells of various lineages, such as melanoma, fibrosarcoma, and lymphoma cell lines. Similar lineage-independent increased anti-tumor responses were also seen in wild-type mice transduced with hepatotropic adeno-associated virus (AAV) coding for mouse sCD6 (smCD6) or given infusions of the recombinant shCD6 protein (43).

### Ligands of CD6

#### What are the functions of CD166/ALCAM?

CD166 or Activated Leukocyte Cell Adhesion Molecule (ALCAM) was the first ligand described for CD6 (44, 45). CD166 is a cell surface glycoprotein and a member of the immunoglobulin superfamily molecules, originally identified on leukocytes. Structurally, CD166 has three domains: a large glycosylated extracellular domain, a transmembrane domain, and a short cytoplasmic domain. The extracellular domain has five immunoglobulin (Ig)-like domains: two amino-terminal membrane distal variable (V)-type domains, and three membrane proximal constant (C2)-type domains. These Ig-like domains mediate homophilic CD166-CD166 interactions and heterophilic CD166-CD6 interactions (46). Subsequent kinetics analyses of CD6-CD166 and CD166-CD166 interactions, done by Hassan et al., revealed that CD6 and CD166 proteins interact with a K_D_ =0.4–1.0 μM and K_off_ ≥0.4–0.63 s^–1^, which is typical of many leukocyte membrane protein interactions. On the other hand, CD166-CD166 homophilic interaction has much lower affinity (K_D_ =29–48 μM and K_off_ ≥ 5.3 s^–1^) (47).

#### Is CD166/ALCAM a therapeutic target in cancer?

CD166 has been found on activated T cells and monocytes, epithelial cells, fibroblasts, neurons, and a wide range of malignances. In autoimmunity, CD166 has been implicated in the pathogenesis of lupus nephritis, rheumatoid arthritis, Sjogren’s syndrome, and inflammatory bowel disease in which both CD6 and CD166 are overexpressed (28, 31, 32, 48). Specifically in cancer, CD166 is associated with worse prognosis and increased metastatic potential chance in several malignancies, including liver (49), thyroid (50), head and neck (51), and breast cancer (52–54). In prostate cancer specifically, CD166-positive cells have enhanced sphere-forming capacity and carcinogenic potential (55), while in breast cancer, CD166 promotes evasion of apoptosis of the cancer cells (56). Furthermore, preliminary results from a phase II study of praluzatamab ravtansine (CX-2009), an activated antibody-drug conjugate that targets ALCAM, show promising overall response rate in breast cancer patients with advanced hormone receptor–positive, HER2 negative cancer (57).

In addition to prostate and breast cancer, CD166 is also up-regulated in liver tissue and serum from patients with hepatocellular carcinoma (HCC) (58). Pancreatic cancer patients whose circulating cancer cells have high levels of CD166, also tend to have significantly shorter survival than those with low levels of CD166 (59). Similarly, high levels of CD166 in colorectal tumors are strictly associated with poorer survival, nodal status, tumor grade and high risk of metastasis (60). Nonetheless, a poor prognosis for cancer is not invariably associated with CD166 expression. High levels of CD166 in patients with Ewing sarcoma are likely to have better prognoses and not develop metastases (61). Similarly, CD166 expression is strongly linked with a better prognosis and longer patient survival in a multivariate survival analysis of patients with non-small cell lung cancer.

The interaction between CD6/CD166 is being studied for its clinical applications in both autoimmunity and cancer (9). Itolizumab, a humanized recombinant IgG1 mAb that blocks the CD6/CD166 binding, is a novel biological agent that has been approved in India for the treatment of psoriasis and psoriatic arthritis (62). Itolizumab downregulates levels of interferon-γ (IFN-γ), IL-6, and tumor necrosis factor-α (TNF-α), leading to reduction in the T-cell infiltration at the sites of inflammation. Itolizumab is currently being evaluated for safety, pharmacokinetics and pharmacodynamics for the treatment of Lupus Nephritis in the U.S. (NCT04128579).

An *in vitro* study suggests that Itolizumab might increase the cytotoxic capacity of CD8 and NK-T lymphocytes to enhance breast cancer cell death. Gonzalez-Munoz et al. demonstrated that such an effect of Itolizumab was due to reversal of the NKG2A/NKG2D ratio and negative modulation of inhibitory CD5 receptor expression on these cell subpopulations, as well as increased granzyme-b and IFN-γ production. Their work also showed the first evidence of the synergistic antitumor effect of combination therapy with itolizumab and pembrolizumab (anti-PD-1). However, the use of CD166 targeted therapeutics for cancer might raise issues of off target consequences related to widespread expression of CD166 on other cell types, including many types of cells of the immune system (63).

Chimeric antigen receptor (CAR) T-cell therapy is emerging as an efficacious cancer treatment for hematological malignancies. Recent pre-clinical studies on CAR-T cells that specifically target CD166 also show substantial promise on various solid tumors. For instance, Wang et al. showed that CD166.BBζ CAR-T cells (a type of CAR-T cells known to persist longer than CD28-costimulated CAR (28ζ) T cells) substantially killed osteosarcoma cell lines *in vitro*. Furthermore, their studies revealed that an intravenous injection of CD166.BBζ CAR-T cells into immunocompromised mice resulted in decreased tumor growth with no apparent toxicity (64).

Similarly, He et al. recently developed CAR-T cells based on the extracellular domain of CD6 and found that such cells are cytotoxic to human colorectal cancer cells (CRC). Interestingly, their studies demonstrated that CD6-CAR-T cells specifically targeted CD166 rather than CD318. Additionally, CD6-CAR-T cells exhibited strong cytotoxicity in a dose-dependent manner against CD166-positive cell lines, and increased production of the cytokine IFN-γ. Perhaps more importantly is the fact that these CD6-CAR-T cells exhibited strong cytotoxicity against CRC cancer stem cells, indicating that CD6-CAR-T might be a promising treatment strategy for CRC (65). Considering that many cancers simultaneously express CD166 and CD318, which bind to different epitopes of CD6, the functional consequences of CD6-mediated interactions between cancer cells and lymphocytes may be a blend of distinct signals from each ligand.

## CD6 and galectins

Galectins are a class of proteins that bind to β-galactose–containing glycoconjugates and play critical roles in developmental, homeostatic, and pathological processes. These proteins, essential in glycobiology, are expressed by fibroblasts, mesenchymal stromal cells, a wide range of cancer cells, activated T and B cells, regulatory T cells, dendritic cells, mast cells, eosinophils, monocytes/macrophages, and neutrophils. In addition, galectins can promote pro- or anti-inflammatory responses, depending on the inflammatory stimulus, microenvironment, and target cells (66–68). In regard to CD6, Escoda-Ferran et al. demonstrated that Galectins 1 and 3 bind to both CD6 and CD166/ALCAM, and interfere with superantigen-induced T-cell proliferation, adhesion, and migration. Moreover, their studies showed that CD6 expression protects T cells from Galectin 1- and 3-induced apoptosis (5). Galectin-1 is considered a pivotal immunosuppressive molecule, and it is expressed by many types of cancer. Tumor-secreted Galectin-1 can bind to glycosylated receptors on immune cells and trigger the suppression of immune cell function in the tumor microenvironment, contributing to the immune evasion of tumors (69). Galectin-3 expression is typically elevated and considered a marker for tumor progression and metastasis, as it is involved in various processes such as cell adhesion, migration, invasion, angiogenesis, and immune suppression, often promoting cancer cell growth and spread across different tissues; essentially, high galectin-3 expression is associated with a poorer prognosis in many cancers (68).

The mechanism by which CD6 expression negatively modulates Galectin 1- and 3-induced T-cell death is not fully understood. Escoda-Ferran et al. demonstrated reduced protection of CD6-negative cells transfected with cytoplasmic tail-truncated CD6 isoforms compared to full-length CD6, suggesting that the down-modulatory effect depends on the integrity of the CD6’s cytoplasmic domain (5). The interaction between galectins and CD6 is thus highly relevant as a potential therapeutic target in various diseases, including cancer.

## Is CD318 important in cancer?

CD318 or Cub domain-containing protein 1 (CDCP1) is a cell-surface glycoprotein expressed by fibroblasts and the epithelium of normal and cancer cells. CD318 is implicated in inflammatory responses, autoimmunity, and cancer. In regard to autoimmune/inflammatory diseases, knockout of CD318 gene has been found to attenuate disease severity and infiltration of IFN-gamma and IL-17-producing T cells in experimental mouse models of encephalomyelitis (6), inflammatory arthritis (29), Kawasaki disease (70), and uveitis (30).

CD318 has been studied almost exclusively in cancer. High levels of cell surface CD318 are associated with progressive disease and markedly poorer survival in breast (71), lung (72), colorectal (73), ovarian (74, 75), renal (76), prostate (77), melanoma (78), pancreatic (79), and hematopoietic cancers (80). This is attributable to the fact that CD318 lies at the nexus of key tumorigenic and metastatic signaling cascades such as the oxidative pentose phosphate pathway, fatty acid oxidation, SRC/PKCδ, PI3K/AKT, WNT, and RAS/ERK axes (81). These signaling cascades play a significant functional role in cancer cell growth and survival, as well as metastasis and resistance to current treatments.

Specifically, CD318 is also known to contribute to the proliferation of breast, lung, ovarian and prostate cancer cells via interacting with HER2 and receptor tyrosine kinases (RTKs), as well as the downstream proteins Ras, Src, and AKT. Alajati et al. demonstrated that the interaction between CD318 and HER2 enhances HER2-driven tumorigenesis and promotes trastuzumab resistance in breast cancer (82). Furthermore, Uekita et al. showed that CD318 is required for the functional link between Ras and Src signaling during the multistage development of human malignant tumors. Further studies demonstrated that, indeed, Ras stimulates the expression of CD318 and promotes Src-mediated survival in *in vitro* lung cancer models of non-small lung carcinoma (83). Similarly, CD318 mediates spheroid growth and is involved in the activation of AKT in ovarian cancer (74). These and other findings have stimulated the development of agents that target CD318 for detection and treatment of a range of cancers, and results from preclinical models suggest that these approaches could be efficacious for the treatment of cancer.

The identification of CD318 as a second CD6 ligand has led to studies that begin to explore its potential role in anti-tumor immunity. Co-culture experiments using breast, lung, and prostate cancer cell lines showed substantial enhancement of cancer cell death in the presence of human lymphocytes and UMCD6, an anti-CD6 monoclonal antibody that rapidly caps and internalizes CD6. Augmentation of lymphocyte cytotoxicity by targeting CD6 is due to direct effects of UMCD6 on NK cells, NK-T cells, and CD8+T cells when CD6 is internalized from the cell surface membrane.

*In vivo*, a single dose of UMCD6 injected intraperitoneally is sufficient to erase CD6 expression from the surface of human lymphocytes adoptively transferred into immunodeficient mice for at least 7 days and thus, maintain their activated phenotype. Further studies confirmed that UMCD6 directly activates the cytotoxic properties of T cells and NK cells by up-regulating the NK activating receptor NKG2D and down-regulating the inhibitory receptor NKG2A (84). *In vivo*, targeting the interaction between CD6 and its ligand CD318 with UMCD6 increases survival of breast and prostate cancer xenografted mice that also receive infusions of human lymphocytes. Analysis of tumor-infiltrating cytotoxic lymphocytes in these mice revealed higher proportions of activated tumor-infiltrating NK cells and CD8+T cells in UMCD6-treated mice compared to IgG and anti-PD-1 control antibodies. Similarly, NK cells treated with UMCD6 showed up-regulation of the NKG2D-DAP10 complex and PI3K pathway (85). These results imply that engagement of CD6 by CD318 weakens the host response against cancer.

Monoclonal antibodies against CD318 have also been studied as a potential approach to cancer immunotherapy. Anti-CD318 produces a more modest effect on cancer cell death and survival compared to UMCD6, which is attributable to a dual effect of UMCD6: 1) rapid internalization of CD6 prevents or reverses engagement of CD6 by its ligand CD318 on cancer cells; and 2) UMCD6 directly activates the cytotoxic properties of CD8+, NK-T and NK cells. Because anti-CD318 does not have any effect on lymphocyte cytotoxic capability, UMCD6 therapy is substantially more effective than anti-CD318 therapy.

In addition, recent pre-clinical work using CAR-T cells containing an anti-CD318 single-chain variable fragment (anti-CD318 scFv), CD3ζ, CD28, and Toll-like receptor 2 (TLR2) domains, shows great promise as a novel strategy for the treatment of colorectal cancer (86). In these studies, Li et al. found that CAR318 T cells exhibited strong cytotoxicity and cytokine-secreting abilities against colorectal cancer cells *in vitro*, and induced cancer regression in xenograft mouse models *in vivo*. Moreover, Schäfer et al. also showed that CAR-T cells specific for CD318 possess strong antitumor capabilities for pancreatic ductal adenocarcinoma (PDAC) (87).

## Does CD6 interact with cancer stem cells?

Importantly, there is now substantial evidence that many cancers are hierarchically organized and driven by a stem-like population that mediates metastasis and treatment resistance (88, 89). Rather than representing a fixed population, “stemness” represents a cell state and “stem-like cells” may arise via de-differentiation of bulk tumor cells. In addition to resistance to radiation and chemotherapy, these cells are also relatively resistant to anti-PD-1/PD-L1 checkpoint inhibition (90, 91). This suggests that increasing the efficacy of immunotherapy as a potential cure of cancers will require effective elimination of this population of cells.

CD166 has been established as a marker for stem cells, particularly in the context of the intestinal stem cell niche, where it is highly expressed on active-cycling stem cells and plays a role in maintaining their function and niche interactions (92). Recent research also indicates CD166 is expressed on hematopoietic stem cells, suggesting its potential as a stem cell marker across different tissue types (93). CD166 has been studied as a potential cancer stem cell marker in many cancers, including colorectal (94), ovarian (95), breast (96), and prostate (97) cancers due to its involvement in cell adhesion.

Similarly, CD318 has been proposed as a stem cell marker as it is also expressed on cells phenotypically identical to mesenchymal stem/progenitor cells (MSCs) and neural progenitor cells (NPCs) (98). Similar to CD166, CD318 expression is also linked to cancer stem cells, indicating its potential role in identifying aggressive tumor cells with stem-like properties in breast cancer (99) and melanoma (78). Further studies are necessary to evaluate the ability of CD6/CD166 and CD6/CD318 directed therapies to target CSCs.

## What is the role of CD44 as a potential CD6 ligand?

CD44 is a cell surface glycoprotein highly expressed on normal stromal cells and on stem cells, in the vast majority of cancers. CD44 binds to several ligands including hyaluronic acid (HA), osteopontin (OPN), chondroitin, collagen, fibronectin, and serglycin/sulfated proteoglycan. Recently, Borjini et al. have identified CD44 as a novel ligand of CD6 (7). Using enzyme-catalyzed proximity labeling and biophysical approaches they demonstrated that CD44 and the other two known CD6 ligands, CD166 and CD318, are distributed diffusely on resting retinal pigment epithelium (RPE) cells but clustered together to form a receptor complex upon CD6 binding. Interestingly, CD6 stimulation dramatically remodeled the actomyosin cytoskeleton in RPE cells, leading to an increase in myosin II phosphorylation. Further studies confirmed that actomyosin activation triggers the disassembly of tight junctions responsible for RPE barrier integrity in a process that required all three CD6 known ligands, providing new insights into the mechanisms by which CD6 mediates T cell–driven disruption of tissue barriers during inflammation.

In addition to RPE, CD44 is highly expressed in many cancers and has a crucial role in regulating metastasis via recruitment of CD44 to the cell surface of the cancer cells. In addition, CD44 is a compelling marker for cancer stem cells of many solid malignancies. The interaction of HA and CD44 promotes EGFR-mediated pathways, consequently leading to tumor cell growth, tumor cell migration, and chemotherapy resistance in breast, prostate, and gastrointestinal cancers (100). In breast cancer specifically, CD44+ cells have superior spheroid colony formation in serum-free medium *in vitro*, as well as enhanced tumor frequency when injected into severe combined immunodeficient (SCID). Moreover, CD44+ gastric cancer cells display similar stem cell properties of self-renewal and are able to give rise to CD44+ cells *in vitro* and *in vivo*. Additionally, these CD44+ gastric cancer cells exhibited increased resistance to radiation and chemotherapy-induced cell death.

Expression of certain vCD44 isoforms is linked with progression and metastasis of cancer cells, as well as poor prognosis. The expression of CD44 isoforms can be correlated with tumor subtypes and also be a marker of cancer stem cells. CD44 cleavage, shedding, and elevated levels of soluble CD44 in the serum of patients is a marker of tumor burden and metastasis in several cancers including colon and gastric cancer (101). In prostate cancer, CD44-positive cells are also capable of enhancing metastasis. Di Stefano et al. demonstrated that CD44v8-10^pos^ cells from PC3 cells are more invasive *in vitro* and have a higher clonogenic potential than CD44^high^ cells (102). Functional consequences of CD44 on lymphocytes binding to CD6 have not yet been examined.

## Are CD6 ligands chemotactic factors?

The first clue that CD6 ligands might be involved in chemotaxis came from Enyindah-Asonye et al., who demonstrated that CD318 is shed from fibroblast-like synoviocytes (FLS) and accumulates in a soluble form in rheumatoid arthritis (RA) synovial fluid at levels higher than found in normal or RA sera. Enyindah-Asonye et al. further confirmed that soluble CD318 is indeed chemotactic for CD6+ lymphocytes at a concentration equal to this *in vivo* gradient. Like FLS, CD318 cancer cells shed concentrations of soluble CD318 proportional to the intensity of CD318 expression on the cell surface. Very high concentrations of chemoattractants, in excess of the optimal concentrations for initiation of chemotaxis, can halt directed migration of cells. Thus, concentrations of CD318+ that are markedly elevated in the vicinity of a CD318+ cancer could potentially halt lymphocytes outside of a tumor and exclude lymphocytes from the tumor microenvironment.

In regard to CD44, Tzircotis et al. demonstrated that hyaluronan acts as a soluble chemoattractant promoting the migration of breast cancer cells *in vitro*. Importantly, they also found that chemotaxis towards hyaluronan can be abrogated upon treatment of the cells with siRNA oligonucleotides to downregulate CD44 expression. Their studies further demonstrated that CD44 is the principal receptor mediating this response, and that CD44 expression is not a general requirement for cell migration and gradient sensing, rather it elicits a ligand-specific response. However, CD44 alone is not sufficient to drive chemotaxis towards hyaluronan, as fibroblasts transfected with high levels of human CD44 do not respond to a hyaluronan gradient (103).

A role for CD166 pathway in chemotaxis has also been reported. Marrocco et al. recently investigated the contribution of CD6 in mediating chemokine-induced migration of T_eff_ cells through endothelial barriers using human PBMCs and found that CD4+ T cells expressing higher levels of CD6 preferentially migrate in response to CXCL12. Blocking the CD6-CD166 pathway with itolizumab reduces the migration of CD4+ CCR7− CD45RA− T_EM_ cells by ~60%. Furthermore, itolizumab treatment reduced the migration of pathogenic TH17 when co-cultured with monocytes.

An important issue in understanding possible roles of CD6 or its ligands in chemotaxis is the paradigm that chemotactic responses are mediated by G-protein coupled receptors (GPCRs). Neither CD6 nor its ligands have the structural attributes of GPCRs. This raises the possibility that an additional CD6-associated protein (or proteins), within the GPCR family but as yet unidentified, is required to concurrently engage ligands of CD6 that generate a chemotactic response.

## Conclusion - the potential of anti-CD6 as a cancer immunotherapy

**Figure 2:**
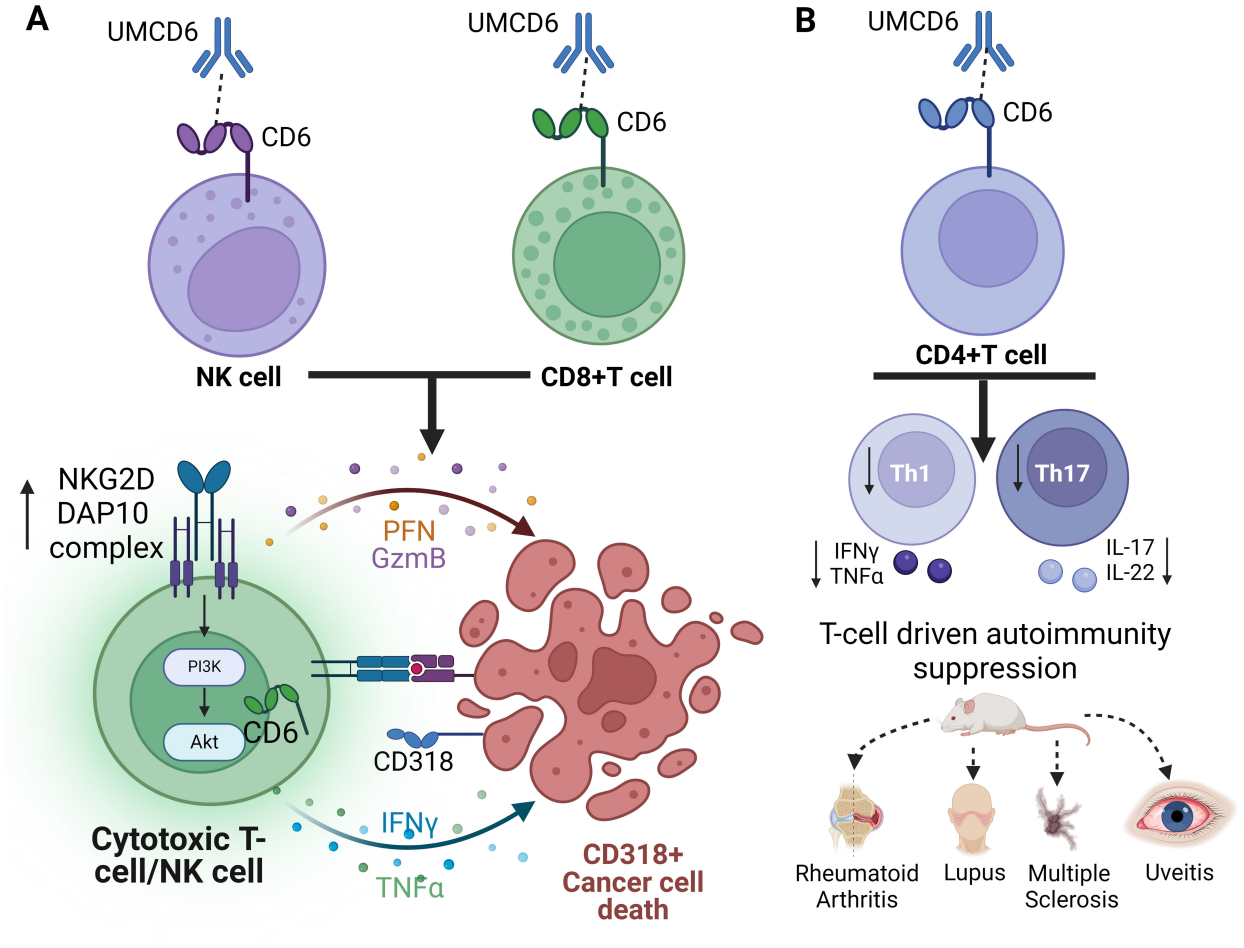
Schematic representation of the dual role of anti-CD6 as a cancer immunotherapy and suppressor of T-cell autoimmunity. **(A)** Internalization of CD6 by UMCD6 leads to increased expression of the activating receptor complex NKG2D-DAP10 and cytotoxic cytokine production on both NK cells and CD8+T cells. **(B)** Internalization of CD6 on CD4+T cells impedes the differentiation of effector Th1 and Th17 cells, offering protection against various mouse models of T-cell autoimmunity.

CD6 ligands have been extensively studied in cancer biology because of their correlation with higher occurrence of metastases, higher relapse rate, and poor prognosis in breast, lung, prostate, colon, melanoma, renal, hepatocellular, acute myeloid leukemia, and pancreatic cancers. However, their role in anti-tumor immunity have just begun to be explored. In this review we highlight a unique dual effect of blocking the CD6/CD318 interaction with UMCD6, an anti-CD6 mAb known to block T-cell dependent autoimmunity through effects on differentiation of effector CD4+T cell subsets, while also activating the anti-cancer cytotoxic properties of CD8+T and NK cells (**Figure 2**). Importantly, current checkpoint inhibitors induce significant autoimmune/inflammatory toxicity in many organs (104–106), which limits the intensity and duration of immunotherapy. Humans or mice that lack PD-1 or CTLA-4 exhibit a global autoimmune diathesis that corresponds to the toxicities observed when these structures are targeted in immunotherapy of cancer. In contrast, *CD6^-/-^* and *CD318^-/-^* mice are healthy and resistant to the induction of autoimmune diseases that are driven by Th1 or Th17 cells. The potential safety of UMCD6 for clinical use is enhanced by lack of depletion of CD6+ lymphocytes by UMCD6, due to its rapid capping and internalization by CD6+ cells, which can avoid engagement of complement or Fc receptors. Although much remains unknown about the interactions of CD6 with its ligands, the data from experimental systems argues for testing anti-CD6 as a novel, lymphocyte-engaging cancer immunotherapy in human cancer patients.
